# Prism Adaptation and Optokinetic Stimulation Comparison in the Rehabilitation of Unilateral Spatial Neglect

**DOI:** 10.3390/brainsci11111488

**Published:** 2021-11-11

**Authors:** Alessio Facchin, Giusi Figliano, Roberta Daini

**Affiliations:** 1 Department of Psychology, University of Milano-Bicocca, 20126 Milan, Italy; g.figliano@campus.unimib.it (G.F.); roberta.daini@unimib.it (R.D.); 2 COMiB—Optics and Optometry Research Center, Università Degli Studi di Milano-Bicocca & NeuroMI—Milan Center for Neuroscience, 20126 Milan, Italy

**Keywords:** unilateral spatial neglect, rehabilitation, prism adaptation, optokinetic stimulation

## Abstract

Prism adaptation (PA) is one of the most effective treatments for the rehabilitation of unilateral spatial neglect. Optokinetic stimulation (OKS) has also been demonstrated to be effective in ameliorating symptoms of neglect. The aim of this study is to compare the effectiveness of these two methods in a group of neglect patients using a crossover design. A group of 13 post-acute brain-damaged patients with unilateral spatial neglect, who had never been rehabilitated, were treated using PA and OKS. Each treatment was applied for 10 sessions, twice a day, to all patients with both treatments in crossed order (i.e., PA followed by OKS or vice versa). Neuropsychological assessments were performed: before the first (T1), at the end of the first/beginning of the second (T2) and at the end of the second training sessions (T3), and two weeks after the end of treatment (T4). Both procedures produced a significant improvement in clinical tests at T2, independent of the type of training. The results suggest that either PA or OKS induces a significant amelioration of neglect in right brain-damaged patients, mainly in the first block of treatment. Since no differences between treatments were found, they could be applied in clinical practice, according to the requirements of the individual patient.

## 1. Introduction

Unilateral spatial neglect designates marked spatial asymmetry in processing spatial information due to brain damage [1]. It is frequently a consequence of right-brain damage as much as a negative predictor of functional outcome [2,3]. Consequently, many rehabilitation procedures have been proposed in order to improve a patient’s condition [4]. Systematic reviews, meta-analyses, and comparisons have shown the levels of efficacy of each type of treatment [5,6,7,8,9,10,11].

The different rehabilitation procedures can be generally divided into the categories of top-down or bottom-up [4]. Top-down therapies refer to different techniques characterized by voluntary and explicit re-orientation of the patient’s attention to the neglected side of space. Bottom-up techniques consist of sensory stimulation that changes the direction of the patient’s attention towards the neglected side of space without any explicit effort [5].

Some studies have applied combined or sequential training in order to boost the efficacy of each method and to improve the overall outcome [12,13,14,15], but very few studies have compared the effectiveness of different treatments. Two bottom-up treatments have been considered here, namely prism adaptation (PA) and optokinetic stimulation (OKS).

Prism adaptation (PA) is one of the most investigated and effective bottom-up techniques among those used for the rehabilitation of unilateral spatial neglect [6,9,16]. It consists of a visuomotor adaptation to rightward prismatic goggles for a short period of time (approximately 20 min), which induces a subsequent realignment of spatial coordinates. Its efficacy in ameliorating many different symptoms of spatial neglect, such as visual exploration of the left hemispace [17,18,19,20,21,22], contralesional somatosensory perception [23,24,25], reading and writing [17,26], wheelchair mobility [27,28], and activities of daily living (ADL) [29,30,31], has been widely reported in the literature. These aforementioned studies report specific and positive outcomes, although some randomized controlled trials (RCT) in USN patients reported limited benefits of this treatment [32,33,34]. The different results found could be ascribed to different patients, testing, treatment, and outcome considered [35]. In fact, meta-analyses based on these mixed results show limited outcomes of PA [36,37].

Optokinetic stimulation (OKS) is a pure bottom-up technique that consists of watching leftward moving visual dots or small squares presented on a standard PC monitor. This stimulation evokes optokinetic nystagmus with the pursuit phase from right to left and fast movement of the eyes in the opposite direction [38]. It does not require the awareness of the deficit by the patient and it has been shown to improve many symptoms of USN syndrome, such as visual exploration in cancellation tasks and auditory neglect [15,39,40], neglect dyslexia [39,41], and the activities of everyday life [42].

Considering the most effective therapies in ameliorating neglect symptoms [5], PA and OKS are the most two effective bottom-up rehabilitation techniques that rely on different explanatory mechanisms. Both therapies involve (directly or indirectly) moving the eyes towards the contralateral side of the lesion and, with this, visuospatial attention, but with non-overlapping mechanisms: the OKS uses horizontal scanning movements and the optokinetic (vestibular) reflex, while the PA concerns the adaptation between different reference systems [43]. If the stimulation of multiple space systems is sufficient, regardless of which ones, then the two treatments could be equally effective. Otherwise, we should find that the most effective treatment that corresponds to the more specific mechanism involved in neglect.

From a theoretical and clinical point of view, PA and OKS are two promising bottom-up rehabilitation techniques that, to our knowledge, have never been compared directly when administered in multiple sessions. Therefore, the aim of this study was to verify whether one of these two rehabilitation methods is more effective than the other, using a crossover design in a group of neglect patients.

## 2. Materials and Methods

### 2.1. Participants

From an initial group of 23 right brain-damaged (RBD) patients who were initially enrolled in the study, only 13 met the inclusion criteria.

The inclusion criteria were as follows: being in the post-acute phase (up to 60 days at the time of the first examination), stroke in the right hemisphere, vascular etiology, being affected by neglect in at least two neuropsychological tests, first-ever cerebrovascular stroke, not had previous neurological or psychiatric diseases, not being subjected to cognitive rehabilitation after the stroke, and choosing to participate in the research.

The restricted selection criteria were based on two principles: (1) patients that have already undergone rehabilitation could ameliorate less than those never treated [14]; and (2) in the chronic phase of deficit, the brain plasticity is almost complete or has reached its limit [11] and consequently poor or no amelioration can be found independently from the type of treatment.

The investigation was performed at the Neuropsychological Service, Rehabilitation Department, Somma Lombardo (IT), A.S.S.T. Valle Olona. Twelve patients were randomly assigned to a group in which PA was performed first, followed by OKS rehabilitation using a crossover design. In the second group, the order of treatment was reversed. Since the baseline level of the two groups was different, the thirteenth patient was not randomized and added to the OKS-PA group following the first neuropsychological assessment.

**Figure 1:**
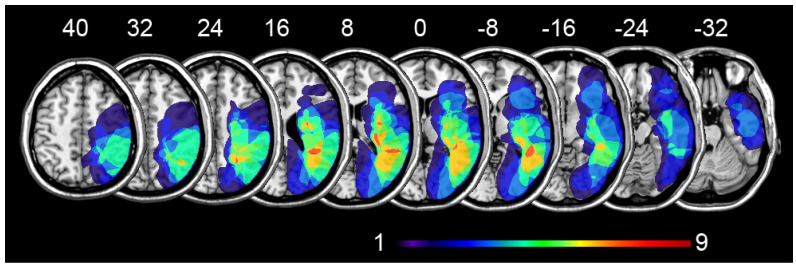
Lesion overlap plots for 12 out of 13 USN patients. Lesions were firstly drawn from CT scans in MRIcron software [45] and subsequently normalized using SPM with clinical toolbox [46]. Brain areas were labeled using the AAL template [47]. MRI axial slices were displayed according to the neurological convention. For each slice, the frequency of lesioned areas is represented through an NIH color scale ranging from 1 to 9 and superimposed on an MNI representative brain template using MRIcron. The procedure of lesion mapping is identical to those already published [48].

The demographic, neurological, and neuropsychological characteristics of neglect patients before any treatment are outlined in Table 1 and Table 2. Imaging data were only available for 12 out of the 13 patients. A normalized representation of the patients’ lesions is shown in Figure 1. The most overlapping damaged areas are the insula, the middle temporal gyrus, and the superior temporal gyrus in the right hemisphere. The white matter tracts involved are the superior longitudinal fasciculus, the external capsule, and the optical tract.

All patients gave their informed consent to participate in the study, in accordance with the ethical standards reported in the declaration of Helsinki, and the approval of the ethics committee was obtained (0065012/15).

### 2.2. Neuropsychological Tests

The neurological and neuropsychological assessment of patients included: mini-mental state examination (MMSE) [49,50], motor, somato-sensitive, and visual field defect (VFD) examination [51], and Brentano illusion test [44] (Table 1).

Since, in USN, dissociations between tests were frequently found [52], the assessment included: letter cancellation, star cancellation, copy drawing, sentence reading, comb and razor, line bisection, clock drawing, and word reading. In order to verify training efficacy, the same battery of tests was used for all four assessments: before the first (T1), at the end of the first/beginning of the second (T2), and at the end of the second training session (T3), and two weeks after the end of treatment (T4). A brief description of these tests is outlined below.

#### 2.2.1. Letter Cancellation

In this A3 size cancellation task, the score is the number of “H” letters canceled by patients (53 on the left side and 51 on the right side of the sheet) within letter distractors. The pathological score for neglect was identified when the left-right difference was at least two targets [53,54].

#### 2.2.2. Star Cancellation

In the A4 star cancellation task, patients were required to circle small stars that were randomly distributed within big stars, with Italian words as distractors [55]. A left–right difference of canceled targets equal to or greater than three is an indication of neglect [56]. The performance of letter and star cancellation tasks was also assessed using the Centre of Cancellation score [57].

#### 2.2.3. Copy Drawing

This task consists of a copy of a five-element complex figure showing: two trees, a house, and two pine trees [58]. According to whether an element in the copy is fully or partially present, each item is scored from 0 to 2, and consequently, the overall score was calculated using a scale from 0 to 10 [59].

#### 2.2.4. Sentence Reading

Patients were required to read aloud six sentences printed in the center of six separate A4 sheets [60]. The number of sentences correctly read represents the score (range 0 to 6). A single error on the left part is considered an indicator of USN [61].

#### 2.2.5. Comb and Razor

This test for personal neglect consists of two parts, comb, and razor (or compact for women). In the section involving a comb, patients were required to pass it through their hair for 30 s, and in that involving the razor (or compact for women), patients were required to shave (or apply make-up) for 30 s. The results were scored using the percentage bias (% bias). The cut-off score for left neglect was >11% [62].

#### 2.2.6. Line Bisection

Patients were required to bisect a series of ten lines 160 mm long and 3 mm thick with their right hand [63]. Each line was centered on a single horizontal A4 sheet. The performance was measured by considering the mean bisection error in mm (deviation from the objective line center, with negative values indicating leftward deviation). A value greater than 4.9 mm is a sign of neglect [44].

#### 2.2.7. Clock Drawing

According to the procedure and scoring methodology described by Mondini (2003) [64], patients were asked to put the numbers and clock hands of a clock in a circle printed on an A4 sheet. Scoring ranges from 0 (no or unrecognizable drawing) to 10 (perfect placement).

### 2.3. Prism Adaptation

The prism adaptation was performed with the terminal exposure procedure [48] using a prism adaptation box (25 cm height, 34 cm depth at the center and 18 cm at the border, and 72 cm width). This box was open on the patient’s and examiner’s sides, curved on the examiner’s side, and flat on the patient’s side. At the border of the curved top panel three targets (pins) were positioned: in the center, at ±21°. The box was used to perform both prism adaptation and open-loop pointing (OLP) measurements. The three targets were used for the adaptation phase, while only the central pin was used for OLP. A transparent vertical panel was positioned on the curved examiner’s side of the box only for the measurement of OLP. A black tissue covered the space between the box and the body of the participants, to avoid the patient from seeing their arm.

The whole prism adaptation procedure comprised three steps.

(1) OLP pre-adaptation. The participant was asked to perform six pointing movements toward the central target from the sternum up to the transparent panel, without prism glasses;
(2) Adaptation. The participant performed the pointing movements with prism glasses, viewing his or her index finger emerging from the examiner’s side of the box for a total of 90 trials;
(3) OLP post-adaptation. Same as point (1) above.

Adaptation was performed with 20^Δ^ (prism diopters, 11.3°) base left prism glasses (Compagnia Ottica Italiana, Milano, Italy). The aftereffect of PA was calculated as OLP post-adaptation minus OLP pre-adaptation measures. The PA procedure was applied as prism adaptation therapy repeating it in different sessions. Patients performed 10 sessions of PA twice a day for five days or within at least 9 days. All data acquired were converted to units of degree (°) in order to allow comparison with previous studies. The prism adaptation procedure was similar to that published by Facchin et al. [25,48].

### 2.4. Optokinetic Stimulation

The OKS stimulation was performed full screen on a 15” 16/10 laptop screen (Toshiba Tecra A10) at about 50 cm of distance. It consisted of 100 black dots of 0.75° diameter randomly presented on a grey background of 16 cd/m^2^ moving from right to left with a speed of 11.3°/s. The procedure lasted 10 min. Patients performed 10 sessions of OKS twice a day for five days or within at least 8 days. The procedure was identical to that reported by Daini et al. [41].

### 2.5. Procedure

**Figure 2:**
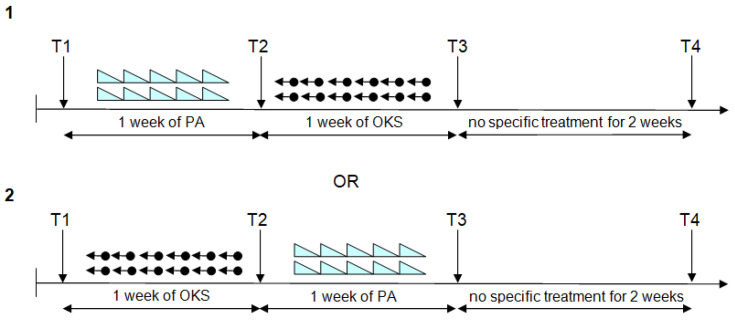
Schematic of the procedure. The two procedures of training, PA and OKS, were applied using a crossover design. **T1**: first evaluation before the first training block; **T2**: second evaluation between the first and second training blocks; **T3**: third evaluation after the second training block; **T4**: follow-up evaluation after two weeks without specific treatment.

A crossover design was applied for the sequence of the two blocks of training. One group of patients performed PA and OKS in this order; the second group performed rehabilitation firstly with OKS and then PA. Each treatment was applied for 10 sessions, twice a day (one in the morning and one in the afternoon) for five days or in the shortest possible time, compatible with the availability and the clinical conditions of patients. Neuropsychological assessments were performed before the first (T1), between the first and the second (T2), and after the second (T3) training blocks, as well as two weeks after the end of treatment (T4). A schematic of the procedure is shown in Figure 2. In order to avoid the short-term direct effect of training, the evaluation from T2 to T4 was performed the day after the last session of rehabilitation. Depending on patient availability and degree of tiredness, the second treatment was able to start after the evaluation. Assessments were performed by A.F. and G.F. Rehabilitation was performed by A.F., G.F., and other clinical staff. A person who differed from the one who made the assessments performed the last treatment. Assessors were aware of the treatment previously performed. During the whole period, patients received physical rehabilitation treatment and occupational therapy. For clinical conditions and availability, the T4 evaluation was obtained only for 8 patients.

### 2.6. Statistical Methods

The aftereffect (AE) of PA was calculated as open-loop post adaptation minus open-loop pre-adaptation and was analyzed using a mixed linear model (MLM) [65,66] with AE size as a dependent variable, session as an independent fixed factor, session as random slope, and patient as a random intercept. Regarding the neuropsychological tests, the patient’s baseline performance for each test was compared between groups at first administration using the Wilcoxon rank-sum test. In this analysis, to avoid false negative, *p*-values were not corrected for multiple comparisons. The outcomes of the different treatments in the two groups were compared using a series of MLM ANOVA with test score as a dependent variable, session as an independent fixed factor, and patient as a random intercept. In order to specifically evaluate the difference between factor levels, MLM-based post hoc comparisons were performed using Holm correction with a significant level of *p* < 0.05.

Following the crossover design, if in the previous analysis, an interaction of Groups x Session was found, a direct comparison of the two methods was performed using the effect of training as post minus pre-session regardless of the position (T3–T2 or T2–T1) for each patient. Statistical analyses and figures were performed with R statistical environment and specific packages [67].

## 3. Results

**Figure 3:**
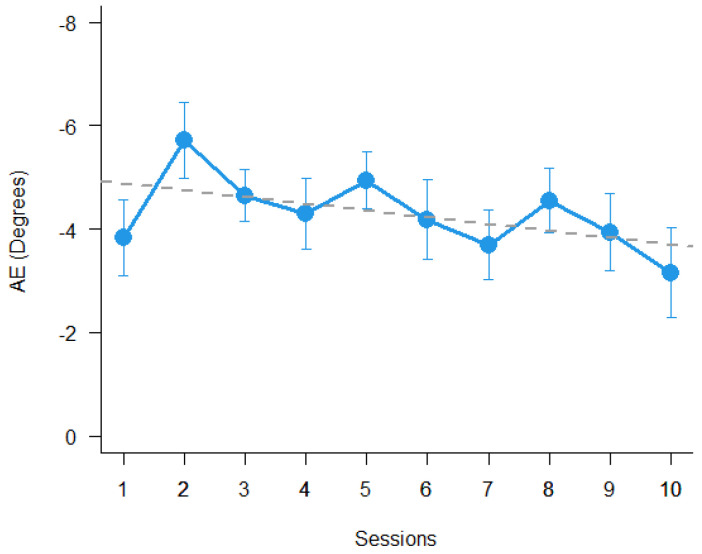
Mean aftereffect of PA over sessions for spatial neglect patients. Bars represent ± 1 SEM.

The results show a significant aftereffect of PA (t _(11)_ = 10.95, *p* < 0.0001), but it did not change significantly over sessions (*p* = 0.12). The results are shown in Figure 3.

**Figure 4:**
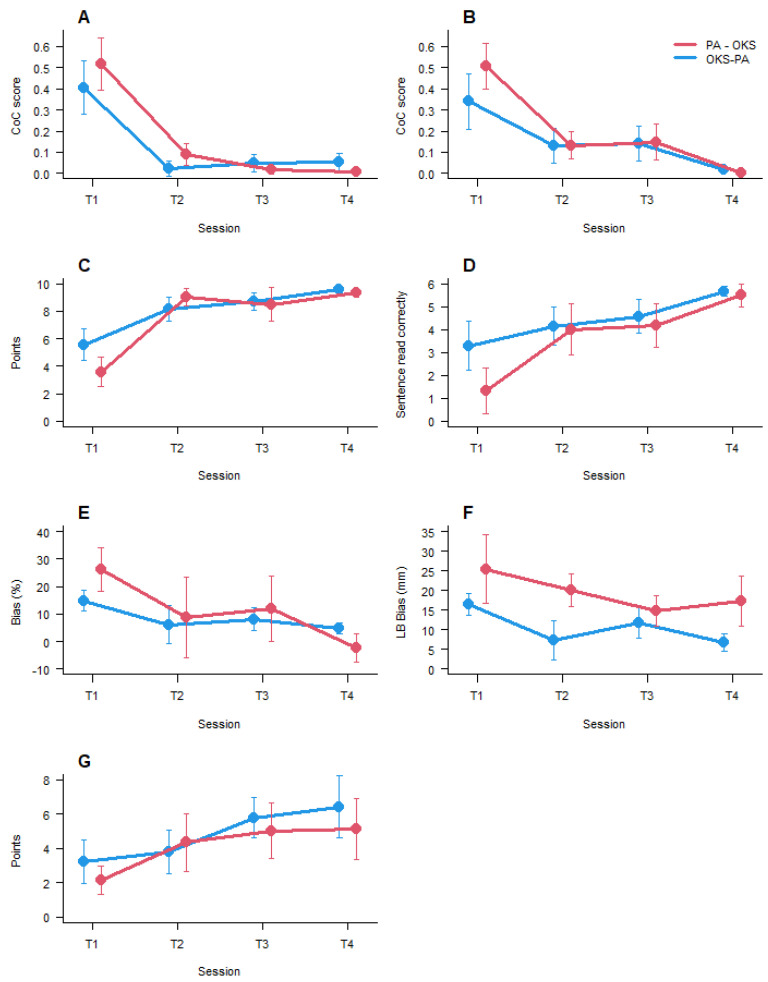
Mean results for each group relative to each specific session. (**A**) = Letter cancellation; (**B**) = Star cancellation; (**C**) = Figure copy; (**D**) = Sentence reading; (**E**) = Comb and razor; (**F**) = Line bisection; (**G**) = Clock Drawing. Bars represent ± 1 SEM. For letter cancellation, star cancellation, comb and razor, and line bisection score, the lower the better. For figure copy, sentence reading, and clock drawing, the higher the better.

The baseline comparison between groups on each neuropsychological test, in the 1st session, at T1, did not reach the appropriate level of significance. The results for the first sessions (T1) are shown in Figure 4.

The results of the comparisons between sessions and groups for all tests showed only a significant main effect of session, specifically for letter cancellation (F _(3, 28.1)_ = 18.43, *p* < 0.0001), star cancellation (F _(3, 27.85)_ = 12.33, *p* < 0.0001), copy drawing (F _(2, 29.95)_ = 21.8, *p* < 0.0001), sentence reading (F _(2, 29.63)_ = 6.7, *p* < 0.005), comb and razor (F _(3, 30.53)_ = 3.14, *p* < 0.05), line bisection (F _(3, 30.39)_ = 3.00, *p* < 0.05), and clock drawing (F _(2, 30.36)_ = 4.5, *p* < 0.01). Post hoc pairwise comparisons showed for letter cancellation, star cancellation, copy drawing, and reading a significant difference between T1 and T2, between T1 and T3, and between T1 and T4. Comb and razor and line bisection Post hoc comparisons showed a significant difference only between T1 and T4. Clock drawing showed a significant difference between T1 and T3 and between T1 and T4. Overall, in all seven tests, there was only a significant main effect of session. In four of seven tests, there was a significant effect of the first training, which was independent of the treatment applied, and there were no significant changes between the 2nd, 3rd, and 4th sessions of testing. In order to demonstrate the absence of any difference, the interaction results for each test, session, and group are plotted in Figure 4.

Since the difference was only found for the main factor sessions, and no interaction between group and session was found, the crossover design did not show a specific difference, and therefore, no further analyses were performed.

## 4. Discussion

The aim of this study has been to compare the effectiveness of PA and OKS applied in multiple sessions as rehabilitation training with a crossover design. As suggested by Tavaszi et al. [11], patients were selected within 60 days after stroke, and, in order to avoid dissociation between clinical and experimental tasks, the same neglect tests were used for inclusion criteria and experimental evaluations [25]. Furthermore, lateralized scoring such as CoC in cancellation tasks and % bias in comb and razor task were used (where applicable) in order to provide a better index of neglect.

It was initially proposed to carry out the rehabilitation procedure twice a day. For various clinical reasons, this frequency was extremely difficult to maintain continuously for 5 days a week. However, the rehabilitation procedure was performed over as short a period as possible, up to within a maximum of 9 days. An interesting question was regarding whether the amplitude of the AE was reducing over sessions. Figure 3 suggests that the visual-proprioceptive AE amplitude tends to decrease over sessions, but this effect is not significant in the sample of USN patients tested.

Overall, in all neglect tests used to evaluate the efficacy of the treatment, there was a main effect of sessions and no effect of group or interactions. This result can signify that there is a strong effect of the first treatment that induces a similar amelioration of symptoms of neglect in the post-acute phase. Indeed, the lack of difference in efficacy after the first session could mean that both types of training are effective in a similar way. No other difference emerged after the first session of training.

The results suggest that the most important aspect for a treatment to be effective is that multiple spatial reference systems are involved (and integrated) [43]. It seems less relevant whether the vestibular or the motor-manual system is involved.

Although a lack of difference in the efficacy of the two types of training could be read as a negative result, it has relevance for clinicians. In a clinical setting, the equivalence of the two interventions allows one or the other method to be prescribed to patients on the basis of their individual needs. If there are some problems with the application of one method, the other could be administered with a similar outcome.

Even if both treatments are bottom up-techniques, they rely on different neural and cognitive mechanisms. Imaging studies in USN patients have revealed that the OKS activates bilaterally frontoparietal regions (FEF and IPS) that have been spared from brain damage and are functionally involved in both oculomotor control and spatial attention [68]. The OKS stimulates the pursuit system [69], and the smooth pursuit eye movements seem to be the key factor of the OKS treatment [70]. Another activation study showed similar bilateral activations of FEF, SEF, premotor cortex, parietal cortex, and, obviously, visual areas. All these areas are extensively activated during OKS compared to passive observation [71].

Concerning PA, there are indications that, in healthy participants and USN patients, PA enhances the representation of visual space in the temporal convexity, the inferior parietal lobule, and the prefrontal cortex of the left hemisphere [72]. These results suggest that, in neglect patients, the left hemisphere provides compensatory mechanisms by assuming the representation of the whole space [72,73]. However, other fMRI studies have demonstrated that there is increased activation in the bilateral parietal, frontal, and occipital cortex (when spared) after PA [74,75].

Overall, these studies indicate that the two treatments rely on some common anatomical bases, but also specific neural correlates.

Regarding the cognitive mechanisms involved, it is interesting that both treatments seem to act on the premotor component of USN. Premotor neglect could be defined as the bias of movements with the ipsilesional arm towards the contralesional side [76,77,78]. Prism adaptation has been shown to have a strong effect on premotor neglect [19,25,77,79], in that there is a great advantage of using this procedure in those tests involving a large motor component. The specific action on premotor neglect could, therefore, also help in suggesting an appropriate explanation for OKS. In fact, the oculomotor component of pursuit eye movement is essentially motor behavior, as much as it is a manual response, and its efficacy could lie in the activation of visuomotor integration. In different ways, both types of training could stimulate the same mechanisms.

Undoubtedly, there are some limitations to this study. Firstly, the sample size of patients is small, primarily due to the strict criteria for inclusion, and the lack of differences could be the result in a low level of statistical significance. However, considering the mean results of patients tested, only a small difference between blocks of training could emerge with a higher sample size. Secondly, there is no control group. This was not planned, because the aim of the study was to compare directly the two procedures and not to perform a more complex randomized controlled study (RCT) on the effectiveness of a therapy. Moreover, the ethical justification for randomizing patients into treatment and not treatment group seems untenable for researching severe brain diseases/injuries. Consequently, the two therapies were compared with one another. The efficacy of the two treatments in the first week and the absence of a difference in the following weeks could also suggest that the spontaneous recovery in the post-stroke critical period is responsible for the improvements observed. A control group could have verified whether this was not the case. Nonetheless, all the literature on PA and OKS suggests that this is very unlikely [23,80,81,82,83,84]. Overall, direct comparison of the two treatments has shown the same relative efficacy in inducing amelioration in a precise post-stroke period of neglect patients. Future studies could also verify the absolute effectiveness of the two trainings using a proper and specific methodology.

## 5. Conclusions

Our results have shown that optokinetic stimulation and prism adaptation are both valid techniques that can be used to improve, in a similar way, the symptoms of spatial neglect in a post-acute phase. Consequently, they could be applied with equal efficacy in clinical practice and by selecting the tests according to the specific requirements of different individuals.

## Figures and Tables

**Table 1:** Demographic, neurological, and neuropsychological characteristics of USN patients at first examination (T1).

Pt. Id	Age	Edu	Sex	Stroke Dist.	Lesion	MMSE	Motor Deficits	Somat. Deficits	VFD	BHT SIE80	BHT SIE160
Pt. 1	82	5	F	55	I	22	+	+	+	25.6 *	58.8 *^,#^
Pt. 2	74	8	M	23	I	20.2	+	−	−	13 *	27 *^,#^
Pt. 3	85	8	M	18	I	19.2	+	+	+	19 *	66.6 *^,#^
Pt. 4	78	5	F	18	H	26.0	+	+	−	27.2 *	42 *^,#^
Pt. 5	73	10	M	21	H	27.9	−	−	+	26 *	21.4 *^,#^
Pt. 6	67	5	F	24	I	24.5	+	+	−	−7.8 *	−6
Pt. 7	75	8	M	18	I	24.2	−	+	+	−4.8	−1.2
Pt. 8	60	11	M	12	I	25.5	+	+	+	32.2 *	30.8 *^,#^
Pt. 9	71	13	M	20	I	19.7	+	+	+	4.8	0.6
Pt. 10	71	11	M	29	I	19.4	+	+	+	2.8	2
Pt. 11	77	5	F	22	H	18.7	+	+	+	6.4	24.2 *
Pt. 12	68	8	M	26	I	23	+	−	+	1.6	−3.6
Pt. 13	58	8	M	34	I	23	−	+	−	15.6 *	27.4 *^,#^

Age and education are expressed in years. Stroke dist. = days between stroke and first neuropsychological assessment. Lesion: I = Ischemic; H = Haemorrhagic; MMSE: scores adjusted for age and education. Motor, somatosensory, and visual field defects (VFD) were evaluated accordingly to the procedures of Bisiach et al. (1983). The Brentano illusion test (BRIT) was administered for distinguishing between hemianopia and pseudo-hemianopia: SIE80 = symmetry of illusory effect for 80 mm illusion; SIE160 = symmetry of illusory effect for 160 mm illusion. * = pathological score [44]. ^#^ = point out certain hemianopia.

**Table 2:** Neuropsychological assessment of unilateral spatial neglect at first examination (T1).

PT. Id	Letter Cancellation	Star Cancellation	Copy Drawing	Sentence Reading	Comb and Razor	Line Bisection	Clock Drawing
Pt. 1	12 *	5 *	1.5 *	0 *	8	12.8 *	2 *
Pt. 2	48 *	24 *	6.5 *	0 *	14.3 *	21.4 *	0 *
Pt. 3	36 *	−2	9.5	5 *	15 *	13.1 *	1 *
Pt. 4	26 *	16 *	4.5 *	2 *	13.1 *	11.6 *	2 *
Pt. 5	11 *	10 *	5 *	6	5.3	23.1 *	7.5
Pt. 6	11 *	2	8.5 *	5 *	17.7 *	9.9 *	1 *
Pt. 7	28 *	23 *	1 *	6	60 *	14 *	1 *
Pt. 8	30 *	13 *	8 *	0 *	12.9 *	15.4 *	8.5
Pt. 9	25 *	1	2 *	6	33.3 *	6.3 *	0 *
Pt. 10	10 *	14 *	1.5 *	0 *	23.8 *	67.3 *	2 *
Pt. 11	23 *	17 *	2 *	0 *	3.9 *	19.3 *	1.5 *
Pt. 12	30 *	20 *	6.5 *	0 *	37.9 *	25.5 *	6
Pt. 13	12 *	18 *	4 *	1 *	16 *	28 *	3 *

Letter and star cancellation = right minus left omissions. Copy drawing = scoring from 0 to 10 (perfect copy). Sentence reading = number of correct sentences read (up to 6). Line bisection = bias in mm from the objective center. Comb and razor = % of bias. Clock drawing = score from 0 to 10 (perfect drawing). * = pathological score according to relative normative studies (see text).

## Data Availability

The data presented in this study are available on request from the corresponding author. The data are not publicly available due to restrictions included in the informed consent provided by patients.
